# The relationship between insulin sensitivity and heart rate-corrected QT interval in patients with type 2 diabetes

**DOI:** 10.1186/s13098-017-0268-3

**Published:** 2017-09-11

**Authors:** Xiao-hua Yang, Jian-bin Su, Xiu-lin Zhang, Li-hua Zhao, Feng Xu, Xue-qin Wang, Xing-bo Cheng

**Affiliations:** 1 Department of Endocrinology, The First Affiliated Hospital of Soochow University, No. 188 Shizi Street, Suzhou, 215006 China; 2 Department of Endocrinology, The Affiliated Haian Hospital of Nantong University, No. 17 Middle Zhongba Road, Haian, 226600 China; 3 Department of Endocrinology, The Second Affiliated Hospital of Nantong University, No. 6 North Hai-er-xiang Road, Nantong, 226001 China; 4 Department of Clinical Laboratory, The Second Affiliated Hospital of Nantong University, No. 6 North Hai-er-xiang Road, Nantong, 226001 China

**Keywords:** Insulin sensitivity, QTc interval, Type 2 diabetes

## Abstract

**Background:**

Reduced insulin sensitivity not only contributes to the pathogenesis of type 2 diabetes but is also linked to multiple metabolic risk factors and cardiovascular diseases (CVD). A prolonged heart rate-corrected QT interval (QTc interval) is related to ventricular arrhythmias and CVD mortality and exhibits a high prevalence among type 2 diabetes patients. The aim of the study was to investigate the relationship between insulin sensitivity and the QTc interval in patients with type 2 diabetes.

**Methods:**

This cross-sectional observational study recruited 2927 patients with type 2 diabetes who visited the Affiliated Haian Hospital and Second Affiliated Hospital of Nantong University. The insulin sensitivity index (Matsuda index, ISI_Matsuda_) derived from 75-g OGTT and other metabolic risk factors were examined in all patients. The QTc interval was estimated using a resting 12-lead electrocardiogram, and an interval longer than 440 ms was considered abnormally prolonged.

**Results:**

The QTc interval was significantly and negatively correlated with the ISI_Matsuda_ (*r* = −0.296, *p* < 0.001), and when the multiple linear regression analysis was adjusted for anthropometric parameters, metabolic risk factors, and current antidiabetic treatments, the QTc interval remained significantly correlated with the ISI_Matsuda_ (*β* = −0.23, *t* = −12.63*, p* < 0.001). The proportion of patients with prolonged QTc interval significantly increased from 12.1% to 17.9%, 25.6% and 37.9% from the fourth to third, second and first quartile of the ISI_Matsuda_, respectively. After adjusting for anthropometric parameters by multiple logistic regression analysis, the corresponding odd ratios (ORs) for prolonged QTc interval of the first, second and third quartiles versus the fourth quartile of ISI_Matsuda_ were 3.11 (95% CI 2.23–4.34), 2.09 (1.51–2.88) and 1.53 (1.09–2.14), respectively, and *p* for trend was <0.001.

**Conclusions:**

Reduced insulin sensitivity is associated with an increase in the QTc interval in patients with type 2 diabetes.

## Background

Reduced insulin sensitivity, which contributes to the pathogenesis of type 2 diabetes, is closely linked to metabolic risk factors and cardiovascular diseases (CVD) [1]. Blunted insulin sensitivity substantially contributes to many metabolic disorders, including central obesity, hypertension, hyperglycaemia, dyslipidemia and atherosclerotic vascular disease [2]. These multiple risk factors may in turn be responsible for a two- to four-fold increase in coronary artery disease and an increase in all-cause and CVD mortality in type 2 diabetes patients compared to the rate in nondiabetic populations [3, 4].

Insulin sensitivity can be quantified with the hyperinsulinemic-euglycemic clamp technique [5]. However, the technique is laborious and is hard to be applied in large-scale clinical or epidemiologic studies. A surrogate measure of insulin sensitivity has been derived from the oral glucose tolerance test (OGTT). The OGTT-based Matsuda index, which is a validated measure of systemic insulin sensitivity, is closely associated with the glucose disposal rate of whole-body during the hyperinsulinemic-euglycemic clamps [5].

The QT interval reflects the total time taken for ventricular myocardial depolarization (QRS complex) and repolarization (T wave). The prolonged heart rate-corrected QT interval (QTc interval) may not only impart ventricular arrhythmias but is also associated with increased all-cause and CVD-related mortality in type 2 diabetes patients [6–8]. A prolonged QTc interval has been shown to be related to various markers of reduced insulin sensitivity, such as abdominal adiposity, hyperlipidaemia, hypertension and hyperinsulinemia [9]. The prevalence of QTc interval prolongation is relatively high in type 2 diabetes patients [10, 11], possibly due to reduced insulin sensitivity. We hypothesize that reduced insulin sensitivity may play a vital role in the prolonged QTc interval in type 2 diabetes, although previous studies have implicated a weak association of insulin resistance with an increased QTc interval in a relatively small sample size of type 2 diabetes patients [12]. If the close relationship between insulin sensitivity and the QTc interval could be demonstrated, strategies targeting to improve insulin sensitivity may ameliorate the prolongation of the QTc interval and improve prognosis in type 2 diabetes patients.

The aim of this study was to explore the relationship between insulin sensitivity assessed by the Matsuda index and the QTc interval from a standard baseline 12-lead electrocardiogram (ECG) in a large Chinese population with type 2 diabetes.

## Methods

### Study design and participants

This cross-sectional study included 2927 patients with type 2 diabetes who were followed-up at the outpatients of the Affiliated Haian Hospital and Second Affiliated Hospital of Nantong University from January 2011 to December 2015. The inclusion criteria were as follows: (1) diagnosis of type 2 diabetes according to the criteria of ADA in 2011 [13] and (2) current use of antidiabetic treatments for more than 3 months. The exclusion criteria were as follows: (1) type 1 diabetes, testing positive for glutamic acid decarboxylase antibody or insulin antibody; (2) type 2 diabetic patients, who presented with instability of glycemic control and high risks of hypoglycemia, and treated with basal-bolus insulin, could not tolerate the OGTT; (3) fibrillation or flutter, atrioventricular blocks, and bundle-branch blocks; (4) heart valve disease, myocardial infarction, and heart surgery; (5) use of any drugs known to affect the QT interval such as tricyclic antidepressants; (6) chronic hepatic disease and kidney disease or malignancy; (7) excessive drinking (alcohol consumption more than 40 g of ethanol daily for women or 60 g daily for men); and (8) acute complications of diabetes, such as hyperglycemic hyperosmolar state and diabetic ketoacidosis; and (9) other endocrine disorders may have effect on glycaemic metabolism, such as hypothyroidism and hyperthyroidism. And informed consents in writing were received from all participants. The study protocol was reviewed and approved by the Medical Ethics Committee of the Affiliated Haian Hospital and Second Affiliated Hospital of Nantong University.

### Baseline data collection

Upon enrolment, all participants were interviewed by trained investigators to record their age, sex, medication use (antidiabetic treatments, hypertensive treatment, and statin medications), health behaviours (smoking and drinking), and medical history of coronary heart disease (CHD). The antidiabetic treatments included lifestyle intervention alone, insulin injection, insulin secretagogues, and insulin sensitizers. CHD was proven by coronary angiography. Body mass index (BMI) was calculated as weight divided by the height square for further analysis. Those with SBP ≥ 140 mmHg, with DBP ≥ 90 mmHg, or receiving antihypertensive agents were considered as hypertensive.

### OGTT procedures and insulin sensitivity index

After an overnight fast, the 75-g OGTT was performed during the early morning. All antidiabetic treatments were withheld at least 24 h before the OGTT. Blood samples were collected at basal, and 30, 60, 120, and 180 min after glucose ingestion for the determinations of plasma glucose and insulin levels. Insulin sensitivity was assessed using the insulin sensitivity index (ISI) proposed by Matsuda and DeFronzo [5]. ISI_Matsuda_ = 10,000/square root of (basal Insulin × basal glucose) × (mean glucose × mean insulin during the OGTT).

### QT interval from electrocardiogram(ECG)

Standard resting 12-lead ECGs (FX-7402, CardiMax, FuTian Beijing Ltd., China) was performed for all participants. The ECG from each participant was recorded on a standard paper with a waveforms-amplitude of 10 mm/mV and a travelling-rate of 25 mm/s. The QT and RR intervals from ten consecutive beats were simultaneously assessed on the ECG. And QT and RR intervals were measured in lead II by two independent experienced physicians who were blinded to the personal information of participants. The QT interval was defined as the duration from the beginning of the QRS complex to the end of the T wave. The beginning of the QT interval was defined as the first negative deflection of the QRS complex, and the end was defined as the point that slope of T wave merged with the baseline [14]. The QT interval was corrected with the RR interval using Bazett’s formula, where QTc (ms) = QT/square root of RR (seconds). QTc interval was the mean of QTc interval from ten consecutive beats. For each participant the QTc interval represented average measurements of the two independent physicians. A QTc interval more than 440 ms was considered abnormally prolonged [9].

### Laboratory examination

Serum insulin level was determined using magnetic beads-based enzymatic spectrofluorometric immunoassay (AIA360, TOSOH). Plasma glucose level (mmol/L) was determined using method of the glucose oxidase (Model 7600 Series, Hitachi). HbA1c concentration was determined by the method of high-performance liquid chromatography (D-10 system, Bio-Rad). Lipid profiles including triglycerides(TG), total cholesterol (TC), low-density lipoprotein cholesterol (LDLC) and high-density lipoprotein cholesterol (HDLC), and serum uric acid (UA) were simultaneously determined by an automatic analyser (Model 7600 Series, Hitachi).

### Statistical analyses

All analyses were conducted using SPSS Statistics V19.0 software (IBM SPSS Inc., USA). Clinical variables were calculated for the total subjects and across the ISI_Matsuda_ quartiles. Continuous variables with normal distributions are presented as the means and standard deviation (SD), whereas skewed distributions were presented as median and interquartile range. Categorical variables were presented as a frequency and percentage. Log-transformations were applied to all variables with skewed distributions for further analyses. The differences in continuous variables between the ISI_Matsuda_ quartiles were compared by One-way analysis of variance (ANOVA), and the categorical variables between the four groups were compared by Chi squared test. The correlation between the log ISI_Matsuda_ and the QTc interval was calculated with Pearson’s correlation test. Multiple linear regression analysis was conducted to compare the influence of the ISI_Matsuda_ and other metabolic factors on the QTc interval. Multiple logistic regression analysis models were also applied to investigate the associations of ISI_Matsuda_ quartiles (Q1–Q3) with the prolonged QTc interval (≤440 vs. >440 ms) relative to Q4, and the odds ratio (OR) and 95% confidence interval (95% CI) were determined. A value of *p* < 0.05 was considered statistically significant.

## Results

### Clinical characteristics

**Fig. 1:**
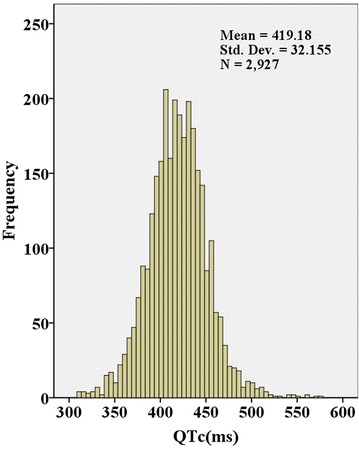
The distribution of the QTc interval

**Fig. 2:**
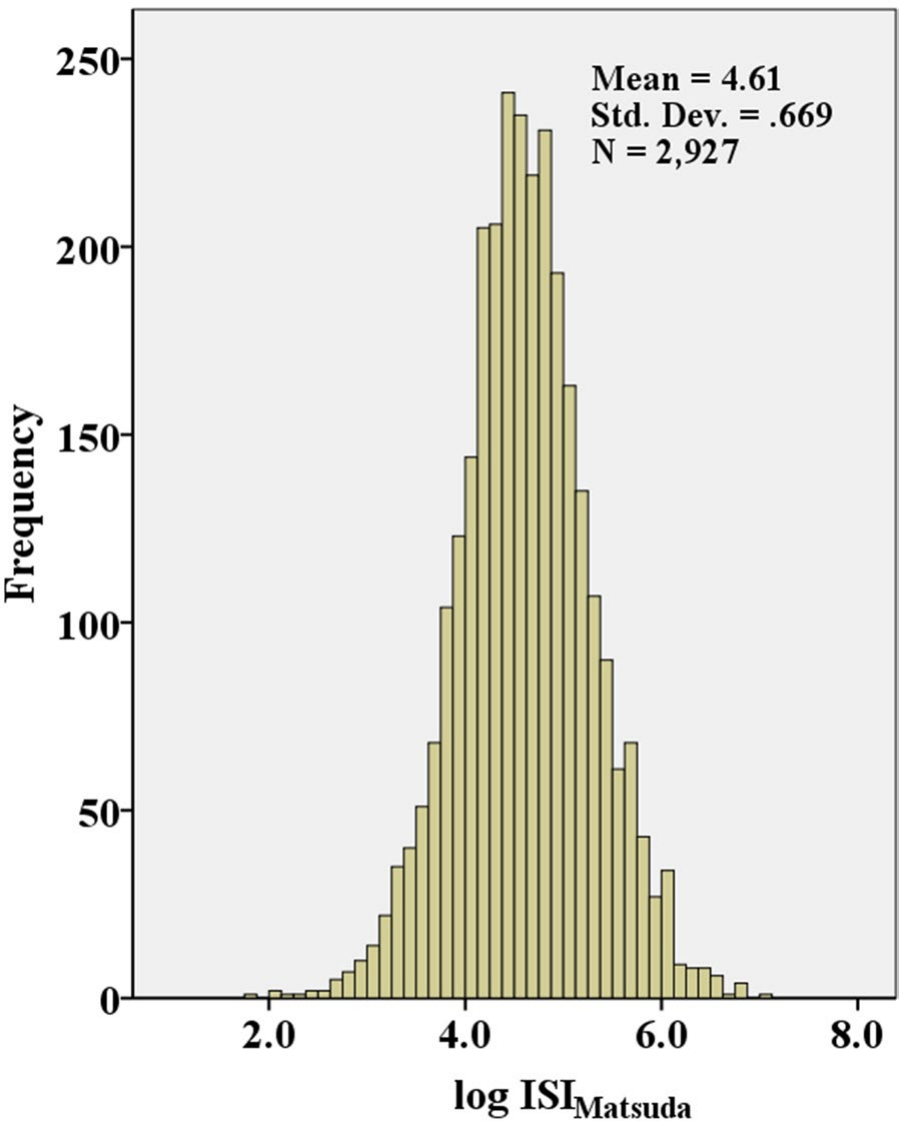
The distribution of the ISI_Matsuda_

The clinical characteristics of the total participants and four subgroups according to the ISI_Matsuda_ quartiles are shown in Table 1, and the distribution of the QTc interval and ISI_Matsuda_ are shown in Figs. 1, 2. The mean QTc interval among all participants was 419 ± 32 ms, and the prevalence of the prolonged QTc interval in our study was 23.4%. The QTc interval significantly increased from 408 ± 31 to 414 ± 31 ms, 423 ± 29 and 432 ± 33 ms from ISI_Matsuda_ Q4 to Q1, and the corresponding proportion of patients with prolonged QTc interval significantly increased from 12.1% to 17.9%, 25.6%, and 37.9% from ISI_Matsuda_ Q4 to Q1, respectively. As the ISI_Matsuda_ quartiles decreased, the ratio of females, BMI, SBP, ratio of hypertension and statin medication, frequency of drinking and CHD, and values of TG, TC, LDLC, serum UA and HbA1c significantly increased, whereas the HDLC level decreased. Age, DBP, diabetic duration and the frequency of smoking did not show differences among the ISI_Matsuda_ quartiles. Comparisons of hypoglycaemic treatments showed that the frequency of insulin treatment increased as the ISI_Matsuda_ quartiles decreased, whereas secretagogues and sensitizers use were comparable among the ISI_Matsuda_ quartiles. The tendency of lifestyle intervention alone was increased when the ISI_Matsuda_ quartiles increased, but the difference was not significant (*p* for trend = 0.067).

**Table 1:** Clinical characteristics of the participants according to ISI_Matsuda_ quartiles

Variables	Total	Q1	Q2	Q3	Q4	*p* for trend
ISI_Matsuda_	99.4 (65.9–151.8)	48.2 (36.9–57.9)	82.0 (73.6–89.8)	120.7 (109.5–135.4)	214.8 (175.0–289.8)	<0.001
log ISI_Matsuda_	4.61 ± 0.67	3.78 ± 0.36	4.40 ± 0.12	4.80 ± 0.16	5.46 ± 0.37	<0.001
*n*	2927	733	727	730	737	–
Age (years)	56 ± 14	57 ± 14	56 ± 14	56 ± 13	55 ± 13	0.280
Female, n (%)	1394 (47.6)	428 (58.4)	368 (50.6)	298 (40.8)	300 (40.7)	<0.001
BMI (kg/m^2^)	25.0 ± 3.9	26.3 ± 4.1	25.5 ± 3.9	24.5 ± 3.6	23.6 ± 3.3	<0.001
SBP (mmHg)	135 ± 17	137 ± 17	136 ± 18	134 ± 17	132 ± 18	<0.001
DBP (mmHg)	80 ± 11	80 ± 10	80 ± 11	80 ± 10	79 ± 11	0.073
Diabetic duration (years)	3.0 (0.3–9.0)	4.0 (0.3–10.0)	3.0 (0.3–10.0)	3.0 (0.3–8.0)	3.0 (0.3–9.0)	0.209
Antidiabetic treatment						
Lifestyle intervention alone, n (%)	313 (10.7)	69 (9.4)	71 (9.8)	79 (10.8)	94 (12.8)	0.067
Insulin treatments, n (%)	871 (29.8)	259 (35.3)	217 (29.8)	198 (27.1)	197 (26.7)	0.031
Insulin-secretagogues, n (%)	1168 (39.9)	255 (34.8)	304 (41.8)	328 (44.9)	281 (38.1)	0.106
Insulin-sensitisers, n (%)	1873 (64.0)	455 (62.1)	470 (64.6)	491 (67.3)	457 (62.0)	0.765
Hypertension, n (%)	1089 (37.2)	338 (46.1)	306 (42.1)	252 (34.5)	193 (26.2)	<0.001
Statins medication, n (%)	1079 (36.9)	303 (41.3)	296 (40.7)	264 (36.2)	216 (29.3)	<0.001
Smoking, n (%)	892 (30.5)	231 (31.5)	232 (31.9)	215 (29.5)	215 (29.0)	0.193
Drinking, n (%)	476 (16.3)	159 (21.7)	131 (18.0)	119 (16.3)	67 (9.1)	<0.001
CHD, n (%)	262 (9.0)	82 (11.2)	74 (10.2)	49 (6.7)	57 (7.7)	0.008
TG (mmol/L)	1.61 (1.02–2.59)	2.06 (1.33–3.20)	1.76 (1.15–2.74)	1.56 (1.00–2.47)	1.15 (0.77–1.86)	<0.001
TC (mmol/L)	4.73 ± 1.25	4.82 ± 1.43	4.81 ± 1.21	4.75 ± 1.19	4.53 ± 1.14	<0.001
HDLC (mmol/L)	1.07 ± 0.29	1.03 ± 0.26	1.06 ± 0.26	1.07 ± 0.30	1.13 ± 0.31	<0.001
LDLC (mmol/L)	2.51 ± 0.82	2.55 ± 0.81	2.54 ± 0.85	2.48 ± 0.81	2.45 ± 0.80	0.021
Serum UA (μmol/L)	286 ± 105	305 ± 104	285 ± 101	285 ± 102	270 ± 108	<0.001
HbA1c (%)	8.29 ± 1.23	8.51 ± 1.32	8.36 ± 1.22	8.23 ± 1.18	8.08 ± 1.14	<0.001
QTc (ms)	419 ± 32	432 ± 33	423 ± 29	414 ± 31	408 ± 31	<0.001
Prolonged QTc, n (%)	684 (23.4)	278 (37.9)	186 (25.6)	131 (17.9)	89 (12.1)	<0.001

Normally distributed values in the table are given as the mean ± SD, non-normally distributed values are given as the median (25 and 75% interquartiles), and categorical variables are given as frequency (percentage)

*ISI*_*Matsuda*_ insulin sensitivity index of Matsuda, *QTc* heart rate-corrected QT, *BMI* body mass index, *SBP/DBP* systolic/diastolic blood pressure, *TC* total cholesterol, *TG* triglyceride, *HDLC* high density lipoprotein cholesterol, *LDLC* low density lipoprotein cholesterol, *Serum UA* serum uric acid, *HbA1c* glycosylated hemoglobin A1c, *CHD* coronary heart disease

*p* values for continuous variables and categorical variables were determined by ANOVA and the Chi squared test, respectively

### Relationship between QTc interval and ISI_Matsuda_

**Fig. 3:**
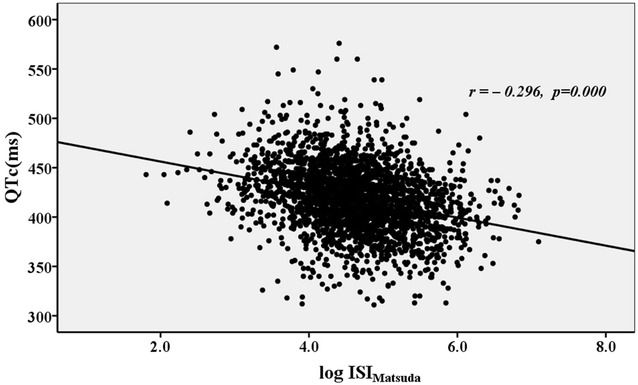
The relationship between the QTc interval and the ISI_Matsuda_

**Fig. 4:**
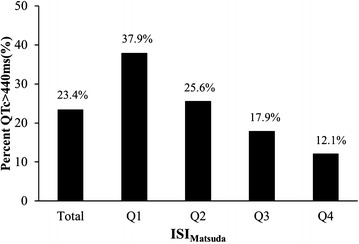
The proportion of prolonged QTc interval (>440 ms) stratified by ISI_Matsuda_ quartiles (*p* for trend <0.001)

The correlation between the QTc interval and the ISI_Matsuda_ is presented in Fig. 3. The QTc interval was significantly and negatively correlated with the ISI_Matsuda_ (*r* = −0.296, *p* < 0.001). The proportion of patients with prolonged QTc interval (>440 ms) increased with the ISI_Matsuda_ quartiles decreased (*p* for trend <0.001) (Fig. 4).

### Multiple linear stepwise regression analysis with QTc interval as the dependent variable

The QTc interval was significantly correlated with the ISI_Matsuda_ in the univariate analysis, and a multiple linear stepwise regression analysis was further performed to assess the associations of the ISI_Matsuda_ and other clinical risk factors, as independent variables, with the QTc interval as the dependent variable for the participants. The independent factors included age, female, BMI, SDP, DBP, diabetic duration, antidiabetic treatments, statins medication, hypertension, drinking, smoking, CHD, TG, TC, HDLC, LDLC, serum UA, and HbA1c. After adjusting for the metabolic risk factors in the multiple linear regression analysis, ISI_Matsuda_, female gender, age, hypertension, insulin treatments and serum UA (*β* = −0.23, 0.22, 0.13, 0.078, 0.062 and 0.059, respectively, *p* < 0.005, total partial *R* ^2^ = 18.4%) were the major independent contributors to the increase in the QTc interval (Table 2), and the ISI_Matsuda_ was the main independent contributor (standardized coefficient *β* = −0.23, *t* = −12.63, *p* < 0.001, partial *R* ^*2*^ = 8.5%).

**Table 2:** Multiple linear regression analysis to explore in dependent risks of QTc interval

Variables	B	SE	*β*	*t*	*p*	*R* ^*2*^
log ISI_Matsuda_	−11.22	0.89	−0.23	−12.63	<0.001	8.5
Female	16.11	1.21	0.22	12.27	<0.001	6.1
Age	0.32	0.045	0.13	7.05	<0.001	2.5
Hypertension	5.10	1.26	0.078	4.05	<0.001	0.7
Insulin treatments	4.23	1.23	0.062	3.44	0.001	0.4
Serum UA	0.018	0.006	0.059	3.056	0.002	0.3

*B* regression coefficient, *SE* standard error, *β* standardized coefficient

### Odd ratios (ORs) of prolonged QTc interval according to quartiles of ISI_Matsuda_

Table 3 also shows the ORs of the prolonged QTc interval according to the ISI_Matsuda_ quartiles. Compared with participants in Q4 of ISI_Matsuda_, the ORs of prolonged QTc interval for Q1, Q2 and Q3 of ISI_Matsuda_ were 4.45 (95% CI 3.41–5.81), 2.50 (1.89–3.30) and 1.59 (1.19–2.13), respectively. After adjustment in the multiple logistic regression, the corresponding ORs of the prolonged QTc interval for Q1, Q2 and Q3 of ISI_Matsuda_ versus Q4 were 3.11 (2.23–4.34), 2.09 (1.51–2.88) and 1.53 (1.09–2.14), respectively.

**Table 3:** ORs for prolonged QTc interval according to ISI_Matsuda_ quartiles (95% CI)

ISI_Matsuda_ quartiles	Q1	Q2	Q3	Q4	*P* for trend
*n*	733	727	730	737	–
Model 1	4.45 (3.41–5.81)	2.50 (1.89–3.30)	1.59 (1.19–2.13)	1-Reference	<0.001
Model 2	4.29 (3.25–5.65)	2.46 (1.85–3.26)	1.59 (1.18–2.14)	1-Reference	<0.001
Model 3	3.58 (2.62–4.88)	2.12 (1.56–2.89)	1.55 (1.13–2.14)	1-Reference	<0.001
Model 4	3.12 (2.24–4.34)	2.08 (1.50–2.87)	1.50 (1.07–2.11)	1-Reference	<0.001
Model 5	3.11 (2.23–4.34)	2.09 (1.51–2.88)	1.53 (1.09–2.14)	1-Reference	<0.001

*Model 1* unadjusted model, *Model 2* adjusted for age and diabetic duration, *Model 3* additionally adjusted for female, BMI, SBP, DBP, drinking, smoking, statins medication, hypertension and history of CHD, *Model 4* additionally adjusted for HbA1c, serum UA, TG, TC, HDLC, and LDLC, *Model 5* additionally adjusted for lifestyle intervention alone, insulin treatments, insulin secretagogues and insulin sensitizers

## Discussion

In the present study, we investigated the association of insulin sensitivity, assessed by the ISI_Matsuda_, with the QTc interval in a large Chinese population with type 2 diabetes. The strengths of our study were follows: first, the prevalence of prolonged QTc interval (>440 ms) was considerably high in this large Chinese population with type 2 diabetes, and the incidence was 23.4%; second, a reduced ISI_Matsuda_ was a major independent risk factor for an increase in QTc interval in type 2 diabetes patients after adjusting for other metabolic risk factors in the multiple regression analysis; third, compared to patients from the fourth ISI_Matsuda_ quartile, those in the third, second and first ISI_Matsuda_ quartiles were associated with an increased risk of a prolonged QTc interval with multiple-adjusted ORs of 1.53 (1.09–2.14), 2.09 (1.51–2.88) and 3.11 (2.23–4.34), respectively.

### The metabolic risks of an increased QTc interval in type 2 diabetes patients

The prevalence of the QTc interval prolongation is considerably high in patients with type 2 diabetes, and many diabetes-related risks may contribute to the increase in the QTc interval. An increased QTc interval may be related to cigarette smoking [15], obesity [16], non-alcoholic fatty liver disease [17], hypertension [18], UA [19], dyslipidemia [20], hyperinsulinemia [21], glycaemic status [22], coronary artery disease [10], carotid intima media thickness [23], diabetic neuropathy [24, 25] and diabetic retinopathy [14]. Meanwhile, reduced insulin sensitivity is the pathophysiologic basis of type 2 diabetes and may underlie above cited risks factors. These risks factors that are associated with decreased insulin sensitivity are the same as those favoring the prolongation of the corrected QT interval. In the present study, insulin sensitivity assessed by the ISI_Matsuda_, insulin treatment, hypertension and serum UA, was significantly associated with an increase in the QTc interval apart from non-modifiable risk factors including age and female. The associations of hypertension, serum UA, age and female with an increased QTc interval were consistent with previous studies. Gastaldelli et al. [26] demonstrated that physiological hyperinsulinemia induced by the euglycaemic insulin clamp acutely prolonged ventricular repolarization as assessed by the QTc in healthy volunteers. Our study revealed that insulin treatment was an independent risk for an increase in the QTc interval. Insulin may prolong the QTc interval in both healthy subjects and diabetic patients. With regard to the relationship between insulin sensitivity and the QTc interval, Shin et al. [27] showed that insulin resistance was an important risk for the prolongation of the QTc interval in normoglycaemic female subjects, and Festa et al. [12] found a weak association of the increased QTc interval with blunted insulin sensitivity, as determined by an intravenous glucose tolerance test in established diabetic patients (*r* = −0.15). Our study revealed that reduced insulin sensitivity assessed by the ISI_Matsuda_ was a major independent contributor to the increase in the QTc interval and accounted for 8.5% of its variation. Strategies targeting to improve insulin sensitivity may provide therapeutic methods to ameliorate the prolongation of the QTc interval and its associated prognosis in type 2 diabetes patients.

### Reduced insulin sensitivity, related risk factors and cardiovascular complications of type 2 diabetes

Reduced insulin sensitivity is the pathophysiologic basis of type 2 diabetes and may underlie a host of metabolic and cardiovascular disorders including glycaemic abnormality, dyslipidemia, hypertension and abdominal adiposity, which comprise the basis of the metabolic syndrome [2]. Each component of metabolic syndrome, which is characterized by reduced insulin sensitivity, is a significant risk factor for CVD. Metabolic syndrome can promote both atherosclerosis and atherosclerotic plaque formation, and the mechanisms possibly involve interaction of the components of metabolic syndrome that promote these processes [28]. Several prospective studies have demonstrated an association between reduced insulin sensitivity and severity of cardiovascular diseases in type 2 diabetes patients [2]. The Verona Diabetes Complications Study by Bonora et al. [29] showed insulin resistance that derived from HOMA was a major predictor for CVD in population with type 2 diabetes. In type 2 diabetes patients, the presence of metabolic syndrome was associated with a nearly fivefold increase in risk of cardiovascular diseases [30]. Our study documented that reduced insulin sensitivity as assessed by the ISI_Matsuda_ was significantly associated with an increase in the QTc interval, which  represented the ventricular myocardial membrane electrical stabilization, in type 2 diabetes patients.

### The possible mechanisms linking reduced insulin sensitivity and the QTc interval

The QTc interval reflects the total time taken for ventricular myocardial depolarization and repolarization, and metabolic, morphological, functional and structural abnormalities of the myocardium may induce ventricular myocardial membrane electrical destabilization and a subsequent increase in the QT interval. In an animal study, Lin et al. [31] observed that obese, insulin-resistant, 16 to 17-week-old rats developed cardiac hypertrophy, exhibited defective inactivation of current, and presented altered electrophysiology characterized by a prolongation of QTc interval. This study suggests that defective calcium inactivation can cause prolongation of the QT interval in patients presented with insulin resistance. Type 2 diabetes is associated with a high prevalence of left ventricular hypertrophy [32], and left ventricular mass is a strong determinant of the QT interval in these patients [9]. Insulin resistance may be important in the development of left ventricular diastolic dysfunction and structure in patients with type 2 diabetes mellitus [33–35]. Insulin resistance and associated hyperinsulinemia in type 2 diabetes can promote the development of a specific form of cardiomyopathy, which is also termed diabetic cardiomyopathy, manifested by left ventricular hypertrophy and diastolic dysfunction [36]. The myocardial triglyceride content is increased in type 2 diabetes patients with insulin resistance [37] and could contribute to concentric remodelling and contractile dysfunction of the left ventricle [38].

## Limitations

It should be noted that our study had several limitations. First, it was a cross-sectional observational study that cannot definitively determine the causality of the association between reduced insulin sensitivity and an increase in the QTc interval. In addition, prospective longitudinal studies are needed to evaluate the cause-effect relationship. Second, this cross-sectional study was performed in a Chinese population, and our findings may lack generalizability to other populations. Third, insulin sensitivity should be assessed by the euglycaemic insulin clamp technique as the gold standard, but this technique was difficult to apply in large epidemiological studies. Insulin sensitivity assessed by the Matsuda index was easily applied in the present large-scale clinical study. Fourth, evaluating insulin sensitivity by ISI_Matsuda_ required endogenous insulin secretion, so this method would not be applicable to diabetic patients with very deficient insulin in response to exogenous glucose. Fifth, although all antidiabetic treatments were withheld at least 24 h before the OGTT, using insulin and insulin sensitizer in diabetic patients may affect the results on OGTT-based insulin sensitivity index. Sixth, we did not analyzed the association of QTc interval with diabetic neuropathy, which may influence the value of QTc interval in the previous studies [24, 25].

## Conclusions

In summary, reduced insulin sensitivity assessed by the Matsuda index is associated with an increase in the QTc interval on standard baseline 12-lead ECGs in type 2 diabetes patients.

## Abbreviations

QTc: heart rate-corrected QT
ISI_Matsuda_: insulin sensitivity index of Matsuda
BMI: body mass index
SBP/DBP: systolic/diastolic blood pressure
TC: total cholesterol
TG: triglyceride
HDLC: high density lipoprotein cholesterol
LDLC: low density lipoprotein cholesterol
UA: uric acid
HbA1c: glycosylated hemoglobin A1c
OGTT: oral glucose tolerance test
CVD: cardiovascular diseases
CHD: coronary heart disease
